# An Overview of Tsetse Fly Repellents: Identification and Applications

**DOI:** 10.1007/s10886-024-01527-5

**Published:** 2024-07-08

**Authors:** Olabimpe Y. Orubuloye, Njelembo J. Mbewe, David P. Tchouassi, Abdullahi A. Yusuf, Christian W. W. Pirk, Baldwyn Torto

**Affiliations:** 1 Department of Zoology and Entomology, University of Pretoria, Private Bag X20, Hatfield, 0028 Pretoria South Africa; 2 Faculty of Infectious and Tropical Diseases, London School of Hygiene and Tropical Medicine, London, United Kingdom; 3 International Centre of Insect Physiology and Ecology (icipe), P.O. Box 30772-00100, Nairobi, Kenya

**Keywords:** African Trypanosomosis, Ecology, Neglected Tropical Diseases, Repellents, Sustainable Development

## Abstract

Tsetse flies are vectors of the parasite trypanosoma that cause the neglected tropical diseases human and animal African trypanosomosis. Semiochemicals play important roles in the biology and ecology of tsetse flies. Previous reviews have focused on olfactory-based attractants of tsetse flies. Here, we present an overview of the identification of repellents and their development into control tools for tsetse flies. Both natural and synthetic repellents have been successfully tested in laboratory and field assays against specific tsetse fly species. Thus, these repellents presented as innovative mobile tools offer opportunities for their use in integrated disease management strategies.

## Introduction

Semiochemicals are chemical signals that mediate intraspecific (pheromones) and interspecific (allelochemicals including kairomones, allomones e.g. repellents, and synomones) interactions among organisms (Nordlund and Lewis 1976; Torto 2009; Norin 2007). Like most hematophagous arthropods, tsetse flies navigate their environment to locate resources (such as hosts, mate, resting and larviposition sites), and reduce mortality-related risks using visual, olfactory, tactile and acoustic cues. However, olfactory cues play crucial roles at long range (Gibson and Torr 1999; Gikonyo et al. 2000, 2003; Olaide et al. 2019). Exploiting the chemicals that mediate these behaviors creates an avenue to manipulate the behavior of tsetse flies and to develop effective control tools.

**Fig. 1:**
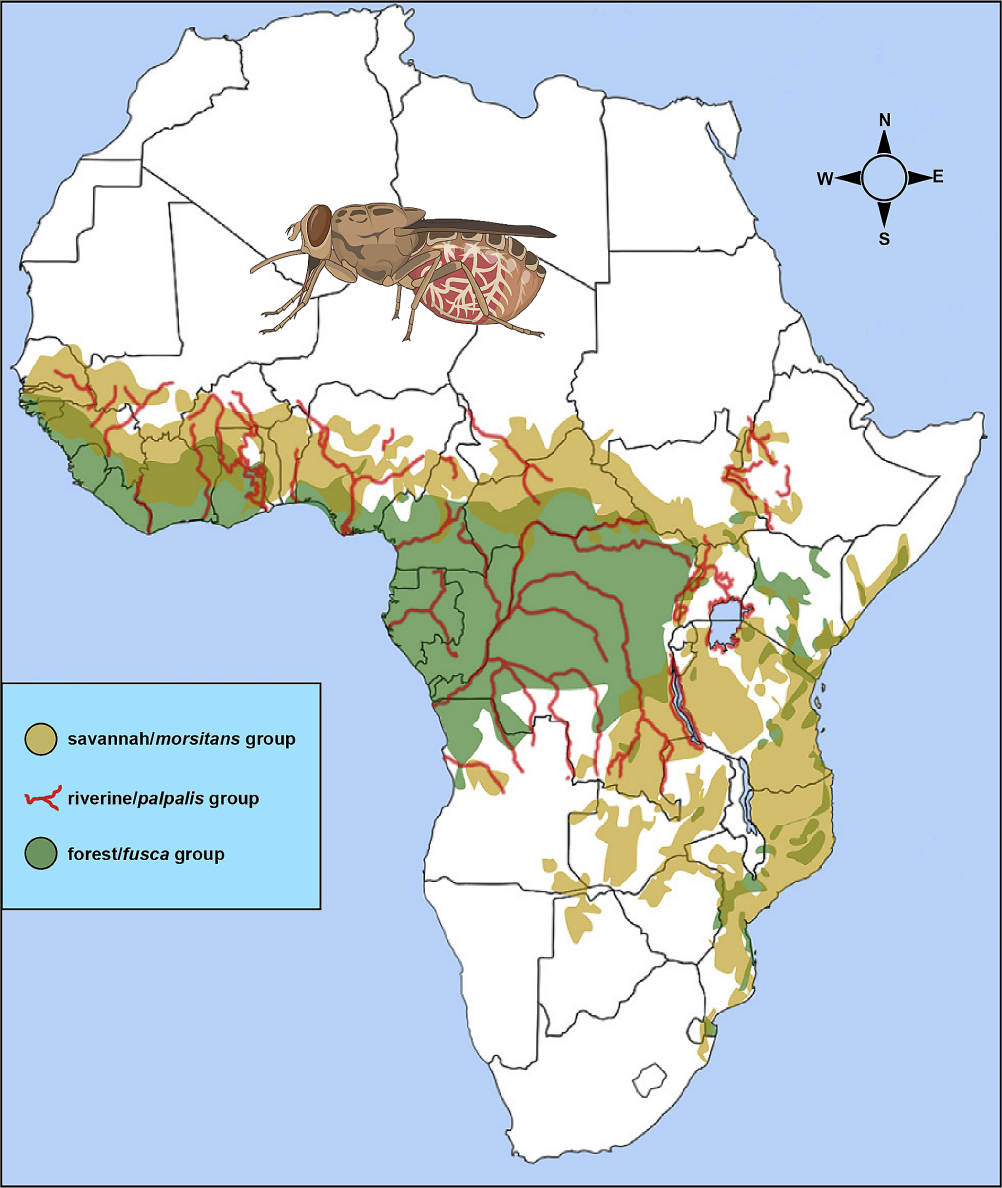
Distribution of savannah, riverine and forest tsetse flies across Africa (Picture modified from Jonas G. King in Krinsky 2019)

Tsetse flies are obligate blood feeding insects found in 37 sub-Saharan African countries (Fig. 1). They are the most important vectors of trypanosome pathogens that cause African trypanosomosis, a devastating neglected tropical disease which affects both humans and livestock (Holmes 2013; FAO and WHO 2022; Vreysen et al. 2013). In sub-Saharan Africa, an estimated 60 million people and about 50 million head of cattle are at risk of infection of the disease-causing pathogens (FAO 2024). African trypanosomosis is a severe constraint to sustainable development in sub-Saharan Africa, particularly in terms of poverty alleviation, food security, good health and wellbeing, and rural development (Alsan 2015; Muriithi et al. 2021). While remarkable progress has been made in eliminating Human African Trypanosomosis (HAT) (FAO and WHO 2022; Franco et al. 2020, 2022), Animal African Trypanosomosis (AAT) is still a serious problem (Abro et al. 2023; Muriithi et al. 2021; Shaw et al. 2017). AAT causes about 3 million cattle deaths per year, and accounts for USD 4.75 billion total annual losses in agriculture and livestock production (FAO 2024).

There are 31 known species and sub-species of tsetse flies placed in three taxonomic groups. They include savannah (or *morsitans*), riverine (or *palpalis*) and forest (or *fusca*) tsetse flies. Nonetheless, only 8–10 species and sub-species are of epidemiological and economic importance (Vreysen et al. 2013). Riverine tsetse flies, notably subspecies of *Glossina fuscipes* and *G. palpalis* transmit the trypanosome pathogens that cause the endemic, chronic and mostly anthroponotic gambian HAT in West and Central Africa (FAO and WHO 2022; Franco et al. 2022; Vreysen et al. 2013). On the other hand, *G. fuscipes* SL together with the savannah tsetse flies *G. swynnertoni*, *G. morsitans morsitans* and *G. pallidipes* transmit the trypanosomes that cause the acute zoonotic rhodesiense HAT in eastern and southern Africa (Franco et al. 2022; Vreysen et al. 2013). Their role in the transmission of AAT depends on geographical location. While riverine tsetse flies transmit the pathogen in West and Central Africa, the savannah group, particularly *G. m. morsitans* and *G. pallidipes* are implicated for transmissions in eastern and southern Africa (Vreysen et al. 2013; Akazue et al. 2019).

Current control of tsetse fly populations includes use of four methods, namely: sequential aerosol techniques, stationary attractive devices (traps and targets), live baits (insecticide treated cattle), and sterile insect technique (Holmes 2013; Vreysen et al. 2013; Gimonneau et al. 2018). However, none of these methods is effective alone, hence their use in tandem (Akazue et al. 2019; Holmes 2013; Musungu et al. 2021; Percoma et al. 2018) and need for novel vector control strategies.

**Fig. 2:**
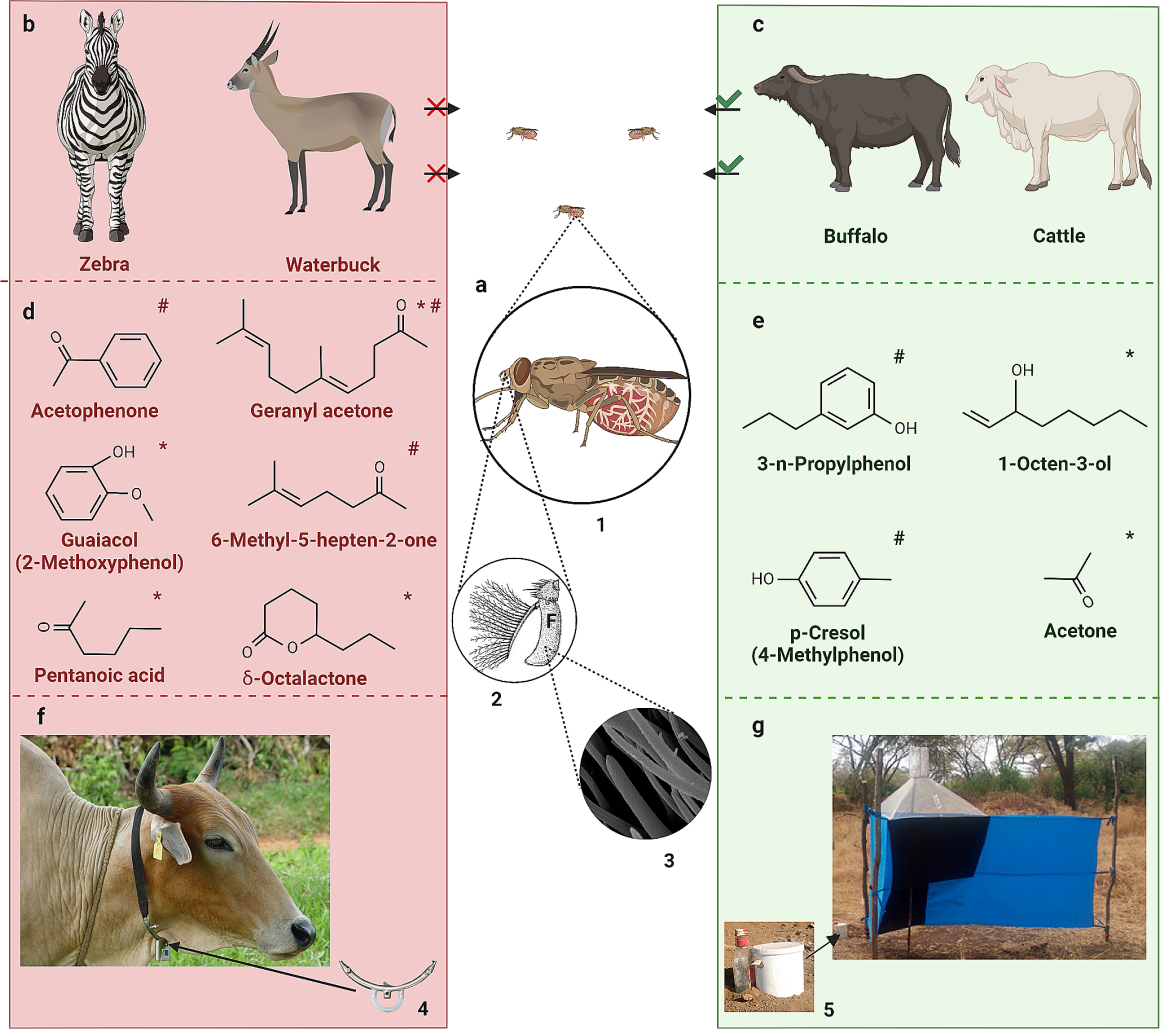
Olfaction-mediated preferential feeding exploited for the control of tsetse flies. (**a**) blood-fed gravid female tsetse fly (**1**) and the antenna (**2**, adapted from Krinsky 2019). The third antennal segment (**F**) is covered with hairlike sensory organs called sensilla (**3**, scanning electron micrograph adapted from Diallo et al. 2020). (**b**) Less-preferred hosts, others include wildebeest, impala and Thompson’s gazelle. (**c**) Preferred hosts, other common ones are warthog, elephant, giraffe, bushpig and bushbuck. Tsetse flies avoid (red x mark) less-preferred hosts such as waterbuck and zebra releasing repellent chemicals while they prefer (green check mark) vertebrates such as cattle and buffalo which release attractants. (**d**) Repellents identified from waterbuck (red asterisk) which are components of the tsetse repellent blend WRB, and components of the zebra-derived repellent blend ZRB (red number sign). (**e**) Attractants identified from Cattle and Buffalo breathe (green asterisk) and urine (green number sign) used as olfactory bait (POCA) in traps and targets for the control of tsetse flies. **(f)** Tsetse repellent technology – Cattle wearing the collar containing the repellent blend WRB identified from waterbuck skin odor (adapted from Saini et al. 2017). The repellent blend is released at predetermined optimum rates from the tubing connected with a repellent reservoir (4) and covered by a protective shield. **(g)** Bait technology – odor-baited Ngu tsetse trap. The host-derived odor bait (5) (cow urine and acetone) attracts tsetse flies at long range beyond the vicinity of the trap, the blue-colored panel of the trap attracts the flies at short range, and the black-colored panel helps to maximize landing. Created with BioRender.com

Semiochemical-based tools developed for tsetse fly control, specifically bait technology, is one of the most successful in the control of hematophagous insects. The obligate hematophagy of tsetse flies and the important role of hosts in mate location (mating usually occur on or in the vicinity of a host (Krinsky 2019) may have contributed to this success. Using a combination of laboratory and field-based behavioral experiments, many attractants have been identified from the breath, skin, and urine of preferred hosts such as buffalo *Syncerus caffer* and cattle *Bos taurus* (Fig. 2c) (Dransfield et al. 1986; Omolo et al. 2009; Owaga et al. 1985; Vale et al. 1986). Subsequently, a four-component attractant blend comprising 3-*n*-propylphenol, 1-octen-3-ol, 4-methylphenol (*p*-cresol) and acetone (POCA) (Fig. 2e), and cow urine and acetone (Fig. 2g) were identified as potent host-derived odor baits (Masiga et al. 2014; Rayaisse et al. 2010; Vale and Torr 2004). The bait technology is efficient for savannah tsetse flies; their combination with traps and targets can increase catches up to ten-fold (Masiga et al. 2014). Likewise, modest extra daily mortality rates of about 2–3% can suppress savannah tsetse fly populations by more than 90% within a period of 12–18 months (Vreysen et al. 2013). However, a major limitation of the bait technology is their immobility which restricts application to small defined areas.

Non-preferred vertebrate hosts of tsetse flies are known to release repellent compounds. These natural repellents and their synthetic derivatives represent another promising semiochemical-based tool that has now been exploited in integrated management of tsetse flies. Here, we review advances in the identification of repellents and development of innovative tools for the control of tsetse flies. Further, we provide insights into the future directions for research in this area.

## Preferential Feeding patterns of Tsetse Flies

Early works analyzing blood meal samples from more than 70, 000 tsetse flies belonging to the three taxonomic groups savannah, riverine and forest collected across many sub-Saharan African countries (Clausen et al. 1998; Moloo 1993; Weitz et al. 1963) clearly revealed certain feeding patterns. Generally, certain vertebrates were more frequently fed on by tsetse flies compared with others (Fig. 2b and c). This host selectivity appears to be important in the ecology of tsetse flies, as the feeding patterns reported in the earlier work have largely been replicated in recent studies (Auty et al. 2016; Channumsin et al. 2021; Ebhodaghe et al. 2021; Gashururu et al. 2023; Kim et al. 2022; Makhulu et al. 2021; Muturi et al. 2011). For instance, in the Serengeti national park Tanzania, the savannah tsetse fly *G. swynnertoni* preferentially fed on warthog, buffalo and giraffe, and *G. pallidipes*, another savannah species, fed on buffalo, giraffe and elephant despite the low densities of these hosts. Interestingly, however, wildebeest, zebra, impala and Thomson’s gazelle which are more abundant were not fed on (Auty et al. 2016). On the other hand, at the Masai Mara National reserve Kenya, African buffalo and elephant were the most common wildlife hosts of *G. pallidipes* and *G. swynnertoni*, and no blood meals were detected from wildebeest and zebra despite their abundance (Makhulu et al. 2021). Similar patterns were reported for the savannah species *G. m. centralis* in Zambia in southern Africa (Gaithuma et al. 2020), and for *G. pallidipes* and *G. m. centralis* in Rwanda, Central Africa (Ghasururu et al. 2023). Despite their ecological relevance, these feeding patterns may be modified or overruled by changes in environment, for example drought, fauna and host availability (Clausen et al. 1998; Hargrove and Williams 1995; Muturi et al. 2011). Additionally, hunger status of tsetse flies and specie-specific preferences (Gikonyo et al. 2000, 2002) may modify these feeding patterns.

Riverine tsetse flies which are mostly opportunistic in their host choice are an exception to these feeding patterns. This exception likely developed as a strategy for survival in their riparian habitat where the probability of contacting a host is low, rather than innate physiological differences compared to savannah tsetse flies (Vale et al. 2014). Macharia et al. (2016) found that genes responsible for transporting hydrophobic molecules, such as the volatile semiochemicals involved in host location, are conserved across the sensillum of both riverine and savannah tsetse flies, supporting the possible ecological basis of the behavioral difference. Moreover, where options are available, riverine tsetse flies show preference for monitor lizards *Varanus niloticus* (Omolo et al. 2009).

The presence of certain allomones (repellent chemicals) emitted in the skin volatiles of less-preferred hosts which are absent or present in trace amounts in preferred ones (Fig. 2b and c) (Gikonyo et al. 2002, 2003; Olaide et al. 2019; Weldon 2010) play a role in the observed feeding patterns of tsetse flies. For instance, certain skin odor components of less-preferred waterbuck (*Kobus ellipsiprymnus defassa*), which are absent in the preferred hosts cattle and buffalo, explain the avoidance of this bovid by tsetse flies (Gikonyo et al. 2002, 2003; Bett et al. 2015). Similarly, the non-preferred zebra releases certain compounds which repel tsetse flies (Olaide et al. 2019, 2021). Other features may offer protection to the non-preferred hosts at close range, for instance, the shaggy coat of waterbuck which make blood feeding difficult (Bett et al. 2015), and the stripes of zebra which usually make landing unsuccessful by confusing most approaching tsetse flies (Britten et al. 2016). However, the repellent chemicals emanating from these non-preferred hosts may have evolved as a first line of defense at long range against blood feeding insects like tsetse flies (Bett et al. 2015; Weldon 2010). While the evolutionary drivers for the feeding patterns are still unclear, the understanding of the feeding behavior of tsetse flies and their contact rates with different hosts have led to the identification of various repellent semiochemicals, which are discussed in detail below.

## Repellents Identified for Tsetse Flies

The use of repellent chemicals to prevent tsetse-host contacts have been suggested since the 1970s, with the identification of lactic acid, acetophenone, hexanoic acid and guaiacol as repellents for *G. pallidipes* and *G. morsitans* (Bursell et al. 1988; Vale 1979, 1980; Vale et al. 1988). Since then, especially in the last decade, research has contributed additional repellents to the existing literature (Table 1).

**Table 1:** Repellents identified for tsetse flies: source, targeted tsetse fly species, collection, analysis and evaluation

Source	Targeted tsetse fly species	Volatile collection method	Method of analysis	Behavioral evaluation	Repellents identified	References
Synthetic^a^	*G. pallidipes* and *G. morsitans morsitans*	-	-	Live host (ox)^b^	Lactic acid (2-hydroxypropanoic acid)	Vale 1979
Synthetic	*G. pallidipes* and *G. m. morsitans*	-	-	Biconical trap, model and electrified netting^b^	Acetophenone and hexanoic acid	Vale 1980
Cattle (*Bos taurus*) urine	*G. pallidipes* and *G. m. morsitans*	Solvent (dichloromethane) extraction	EAD, GC, GC/MS	Wind-tunnel bioassay^c^, Beta or F3 traps and targets^b^	Guaiacol (2-methoxyphenol)	Bursell et al. 1988; Vale et al. 1988
Synthetic	*G. pallidipes*	-	-	Epsilon traps and live host (ox)^b^	Acetophenone, pentanoic acid, hexanoic acid and guaiacol	Torr et al. 1996
Waterbuck (*Kobus ellipsiprymnus defassa*) skin	*G. pallidipes* and *G. m. morsitans*	Adsorption onto activated charcoal and reverse-phase silica, elution with dichloromethane	GC/EAD, GC, GC/MS	Two-choice wind tunnel assay^c^, Ngu trap and live host (ox)^b^	Pentanoic acid, hexanoic acid, heptanoic acid, 2-octanone, 2-decanone, 2-undecanone, 2-dodecanone, geranyl acetone, guaiacol, carvarol and δ-octalactone	Bett et al. 2015; Gikonyo et al. 2002, 2003
Synthetic	*G. pallidipes* and *G. m. morsitans*	-	-	Two-choice wind tunnel assay^c^, Ngu trap and live host (ox)^b^	4-Methylguaiacol^d^	Saini and Hassanali 2007
Synthetic	*G. pallidipes* and *G. m. morsitans*	-	-	Two-choice wind tunnel assay^c^, Ngu trap^b^	δ-Nonalactone^d^	Wachira et al. 2016
Synthetic	*G. fuscipes fuscipes*	-	-	Biconical trap and sticky small targets^b^	Pentanoic acid, guaiacol, δ-octalactone, geranylacetone and 4-methylguaiacol	Mbewe et al. 2019
Zebra (*Equus quagga*) skin	*G. pallidipe*s and *G. f. fuscipes*	Dynamic headspace volatile collection onto adsorbent filter (Carbopak B), elution with dicholoromethane	GC/EAD, GC/MS	Ngu trap^b^	6-Methyl-5-hepten-2-one, acetophenone and geranyl acetone	Olaide et al. 2019, 2021

^a^ Lactic acid is a component of human skin odor which may account for the repellence of natural human skin odor to *G. pallidipes and G. m. morsitans* (Vale et al. 1979)

^b^ Laboratory-based experiment; ^c^ Field-based evaluation

^d^ 4-Methylguaiacol and δ-nonalactone are derivatives obtained by structural modification of the natural guaiacol and δ-octalactone,^,^ respectively

Investigation of the possible semiochemical basis to explain the preferential feeding patterns of tsetse flies on different vertebrates led to the identification of natural repellents. In their pioneer work, Gikonyo et al. (2000) observed that caged individuals of *G. m. morsitans* showed significantly higher reluctance to feed on the less-preferred waterbuck or on waterbuck sebum-smeared feeding membranes, compared to the preferred ox or untreated feeding membranes. This suggests an allomonal basis for the differential feeding of tsetse flies on these bovids in the wild. Follow-up coupled gas chromatography-electroantennographic detection (GC/EAD) and GC-mass spectrometry (GC/MS) studies comparing the responses of *G. m. morsitans* and *G. pallidipes* to the skin odors of waterbuck and preferred hosts (ox and buffalo) identified fifteen compounds. These included straight chain C5 - C10 carboxylic acids, C8 – C12 2-ketone homologues and geranyl acetone, phenols (guaiacol and carvarol), and a lactone (δ-octalactone) (Gikonyo et al. 2002). These compounds which repelled *G. m. morsitans* in a laboratory two-choice wind tunnel assay were either specific to waterbuck alone or only present in trace amounts in the preferred hosts (Gikonyo et al. 2003). This finding was interesting and confirms that the identified semiochemicals underpin the preferential feeding of tsetse flies on these vertebrates. Subsequent field studies by Bett et al. (2015) reduced the complex fifteen-component blend comprised of four classes of compounds (acids, ketones, phenols and δ-octalactone) to a five-component (guaiacol, geranyl acetone, hexanoic and pentanoic acid and δ-octalactone) repellent blend. Subtractive assays showed significant reduction in trap catches (84%) and feeding efficiency on ox (96%) relative to the control. The recorded reduction in trap catches and feeding efficiency by the five-component blend was comparable to the fifteen-component blend (90% and 94%, respectively) (Bett et al. 2015). Based on redundancy, hexanoic acid was later removed from the blend (Saini et al. 2017), leaving a four-component tsetse fly repellent blend named waterbuck repellent blend (WRB) (Fig. 2d).

Following this finding on the repellents mediating avoidance of waterbuck by tsetse flies, Olaide et al. (2019) investigated the semiochemical basis of the avoidance of zebra, another less-preferred host of tsetse flies (Fig. 2b). In their study, crude zebra (*Equus quagga*) skin odors significantly reduced field Ngu trap catches of *G. pallidipes* (66.7%) compared to attractant-baited traps. This indicated that like in the waterbuck, repellent semiochemicals released by zebra contributed to their avoidance by tsetse flies. GC/EAD and GC/MS analyses identified seven electrophysiologically-active components as candidate repellents from the crude skin odors. These components included 6-methyl-5-hepten-2-one, acetophenone, geranyl acetone, heptanal, octanal, nonanal and decanal. In field studies, Olaide et al. (2019) found that, a seven-component blend of these compounds mimicking their natural ratio of occurrence in zebra skin odor significantly reduced catches of *G. pallidipes* (48.9%). This compared with the crude skin odor and the repellency of WRB (58.1– 59.2% catch reduction). Remarkably, further subtractive assays showed the repellency of the crude zebra skin odor and the seven-component blend was due mainly to the three ketones 6-methyl-5-hepten-2-one, acetophenone and geranyl acetone (62.7% catch reduction) (Fig. 2d) (Olaide et al. 2019). This resultant three-component blend ZRB, comprised of only ketones, which may be more stable compared to WRB, and an excellent alternative repellent blend for tsetse flies which may be easier to formulate for large-scale use. Further, these results should encourage research into possible semiochemical basis of the avoidance of other non-preferred hosts of tsetse flies.

Apart from investigating other less-preferred hosts, structural modification has also proved useful in the identification of new tsetse fly repellents. For instance, derivatization of the natural repellent guaiacol by replacing hydrogen in the “4-” position with a methyl substituent (4-methylguaiacol) significantly increased repellency to *G. m. morsitans* in 2-choice wind tunnel assays (Saini and Hassanali 2007). The 4-methyl derivative also significantly reduced numbers of *G. pallidipes* attracted to traps and to ox odor, and the proportion that fed on ox by > 80%. Similarly, increasing the side chain length of the repellent δ-octalactone from -C_3_H_7_ to -C_4_H_9_ (δ-nonalactone) enhanced repellency to *G. pallidipes* and *G. m. morsitans* in laboratory assays, and to *G. pallidpes* in field studies (Wachira et al. 2016). A four-component blend of these two synthetic analogues (4-methylguaiacol and δ-nonalactone), and heptanoic acid and geranyl acetone, representing the four classes of compounds in the original WRB (Bett et al. 2015; Saini et al. 2017) was found to be promising as a repellent for savannah tsetse flies (Wachira et al. 2020). However, the repellency of this hybrid blend (natural repellents and structurally modified derivatives) compared to the natural blend WRB, and effects on feeding efficiency and disease incidence are still unknown.

The development of odor-based tools for the control of the riverine tsetse flies, which are responsible for more than 97% of reported HAT cases and important in the transmission of AAT (Opiro et al. 2017; Tirados et al. 2015), have been challenging. This is because of the perceived low responsiveness of these species to odors (Oloo et al. 2014; Torr and Vale 2015; Vale et al. 2014). Recent findings, however, have shown that odor cues play key roles in their ecology (Mbewe et al. 2019; Olaide et al. 2021). For instance, results from a study conducted on four islands of Lake Victoria in Kenya showed a significant reduction in catches of the riverine tsetse flies *G. fuscipes fuscipes* in biconical traps and sticky small targets in the presence of WRB and 4-methyl guaiacol compared to control trap or target alone (Mbewe et al. 2019). Likewise, field evaluations of the repellent blend identified from zebra skin odor (ZRB) on *G. f. fuscipes* in the same study area showed significant reductions in biconical trap catches similar to the WRB (Olaide et al. 2021). Although the observed repellency of WRB and ZRB on *G. f. fuscipes* was lower compared to previously reported data for savannah tsetse flies (Mbewe et al. 2019; Olaide et al. 2021), chemosensory gene families responsible for host selection in these two tsetse fly species appeared to be conserved across the sensilla of both fly groups (Macharia et al. 2016). Therefore, the observed variation in repellency may relate to ecological adaptations in the different habitats of the savannah and riverine tsetse flies as previously suggested (Omolo et al. 2009; Vale et al. 2014). Regardless of the lower repellency, these results indicate the potential for the application of the repellent blends WRB and ZRB initially identified for savannah tsetse flies in the control of riverine tsetse flies and integrated management of HAT.

Individual compounds in a repellent blend may differ in their relative contribution to repellency or interaction with the olfactory system of tsetse flies at the molecular and cellular level. For instance, of the four components of WRB, geranyl acetone is a major contributor to the repellency of WRB to both the savannah *G. pallidpes* (Bett et al. 2015) and the riverine *G. f. fuscipes* (Mbewe et al. 2019). Further, geranyl acetone has been found to contribute significantly to the antifeedant effect of the repellent blend WRB (Diallo et al. 2020). Interestingly, geranyl acetone is the only shared component between WRB and the recently identified ZRB (Fig. 2d), an important component of ZRB eliciting repellency in both tsetse fly groups (Olaide et al. 2019, 2021). In fact, when the components of ZRB were tested individually, geranyl acetone alone replicated the repellency of ZRB on *G. f. fuscipes* (Olaide et al. 2021). By contrast, certain components may be only marginally repellent when tested alone, however, they synergize activities of other compounds. For example, 6-methyl-5-hepten-2-one alone had minimal and no significant effect on trap catch reduction of *G. pallidipes* (Olaide et al. 2019)d f. *fuscipes* (Olaide et al. 2021), respectively. However, when combined with other components of ZRB (acetophenone and geranyl acetone), it significantly increased repellency, up to 50% for *G. f. fuscipes* (Olaide et al. 2021).

Investigating the mechanisms of detection and coding of these chemicals on tsetse fly antennae may unravel the sensory basis of the observed differences in their contribution to the repellency of the blends. Further, this may enhance effectiveness of existing repellent blends or lead to the development of novel blends. In their study, Diallo et al. (2020) predicted the olfactory receptors (ORs) in *G. f. fuscipes* antenna responsible for coding the individual components of WRB (Fig. 2d) and evaluated the effect of the individual components on their mRNA transcripts. While all the components produced a mixture of up and down regulations of the mRNA transcripts, there was a correlation with their antifeedant effect. Strong antifeedants such as geranyl acetone induced up- and down-regulation in almost equal number of OR mRNA transcripts, while guaiacol which had no observed effect on feeding inhibition upregulated mRNA transcripts of more ORs (Diallo et al. 2020). Considering its commonality in both repellent blends (WRB and ZRB), high spatial repellency and strong antifeedant activity, geranyl acetone may play important roles in the ecology of tsetse flies. Thus, it should be considered as a potential candidate single component tsetse fly repellent which would require additional research. Additionally, subject to further study, structural modification of the natural geranyl acetone may reveal derivatives that are equally or more effective repellents.

### Application of Host-Derived Repellents in Tsetse fly Control (Tsetse Repellent Technology)

Apart from the vector control methods that aim to reduce tsetse fly populations such as attractant-baited traps (Fig. 2g) and targets, tactics that limit vector-host contact, such as the use of repellents, can disrupt disease transmission cycle and incidence (Saini et al. 2017). One technique by which repellents have been applied in vector control and disease management is in the use of innovative repellent collars from which the repellent blend WRB is dispensed at controlled release rates (Fig. 2f). In a field trial in coastal Kenya involving 1,100 cattle, these repellent collars worn by individual cattle under natural tsetse fly challenge (savannah tsetse flies predominantly *G. pallidipes*) provided significant protection against disease incidence (> 80% reduction) (Saini et al. 2017). The innovative repellent collars (Fig. 2f) protect members of the herd against tsetse fly bites, allowing free-grazing in areas infested by tsetse flies, either alone or combined with insecticide treated traps in a “push-pull” approach. Immediate farm-level socioeconomic benefits included increase in herd size, market value, land cultivation, improved food security and household income (Saini et al. 2017; Muriithi et al. 2023). Further, the use of the repellent collars compared favorably with trypanocides in terms of costs, and significant reduction (> 60%) in trypanocide use (Saini et al. 2017). Subsequent expert elicitation survey from 18 countries across different regions of sub-Saharan Africa estimated a benefit: cost ratio of the WRB as 9:1 and monetary gains of US$ 78–869 million per annum (Abro et al. 2021). These data show that the tsetse repellent technology represent an environment smart, sustainable and cost-effective tsetse fly and African trypanosomosis management approach.

Key strengths of the tsetse repellent technology are its mobility and ease of use which are compatible with the pastoralist lifestyle of livestock keepers in sub-Saharan Africa. In addition, unlike stationary traps (Fig. 2g) and targets which are considered public goods, tsetse fly repellent collars (Fig. 2f) are owned and maintained by the livestock keepers (Saini et al. 2017). The limitation of repellents is that their efficacy is dependent on the population density of tsetse flies and disease incidence (Torr et al. 2011). As such, application of repellents to protect cattle in areas with high tsetse fly challenge or disease incidence will require integration with other vector control methods that reduce tsetse fly population such as insecticide treated traps and targets. Encouragingly, the tsetse repellent technology might be more effective in the integrated management of riverine tsetse flies and elimination efforts of HAT because of the characteristic low infection rates of the causal pathogenic *Trypanosoma brucei* species. Here, the repellents could be incorporated into clothing, necklaces and hand bands as personal protective materials.

## Future Perspectives and Conclusion

Tsetse flies and African trypanosomosis are at the focal point of poverty, hunger, and poor health and wellbeing in sub-Saharan Africa. Given the challenges in developing a successful vaccine, and the ineffectiveness and toxicity of available trypanocides (Delespaux and Koning 2007; FAO 2022; Meyer et al. 2016), integrated vector control approaches are likely to be more effective in disease management. However, limitations of available control methods for tsetse flies, including sequential aerosol techniques, traps and targets, live baits and sterile insect technique, motivate ongoing studies to develop new innovative vector control methods. Semiochemical-based or assisted control tactics have been useful in this regard, specifically host-derived attractants used as baits in traps and targets and recently, the tsetse repellent technology.

There is immense opportunity for improvement of the newly developed tsetse repellent technology. The design of combination tactics such as “push-pull” informed by using predictive models to understand the distribution of the semiochemical plumes and flight dynamics of tsetse flies under different field conditions may enhance efficacy. For instance, in livestock, members of the herd wearing the repellent collar could “push” flies away and those not wearing repellent collar but treated with insecticide may act as a dead-end “pull”. Alternatively, flies that are “pushed” away from hosts by the repellent blend may be “pulled” into an attractant-baited trap or target in a more area-wide approach, especially when there is high disease incidence or tsetse fly challenge. In both instances, reduced use of insecticide may help mitigate development of resistance to insecticides, and other environmental and ecological concerns associated with insecticide use. Additionally, if treating only selected members of a cattle herd (for instance males which are more attractive compared to females and calves (Torr et al. 2006, 2007) with the repellent collars could protect the whole herd, then the costs will be significantly reduced and research into this possibility is warranted. Apart from the above, comparison of the two repellent blends WRB and ZRB (in terms of efficacy in reducing feeding efficiency and disease incidence in the field, stability/longevity, and cost) and studying possible synergy between these blends for an enhanced repellency will also be useful. Finally, apart from waterbuck and zebra, the possible semiochemical basis of the avoidance of other less-preferred hosts of tsetse flies such as wildebeest and impala is still unknown. Apart from filling a fundamental gap in knowledge, investigating this possibility might reveal identical or novel and equally potent repellents for tsetse flies.

It has been reported that infection with disease-causing organisms can change the chemical profiles of vertebrate hosts (Emami et al. 2017; Getahun et al. 2022a; Magalhães-Junior et al. 2014; Peled et al. 2012; Shirasu and Touhara 2011) and disease vectors (Ebrahim et al. 2023). As such, it is important to investigate how trypanosome infection could alter odor profiles and attractiveness of less-preferred vertebrates to tsetse flies. Apart from revealing potential unknown aspects of tsetse fly ecology and disease epidemiology, such investigations could also lead to the identification of novel semiochemicals (attractants and repellents) to be explored for tsetse fly control.

Characterizing the physiological, cellular and molecular basis of odor detection, coding and processing in the tsetse fly antennae leading to different behavioral responses will facilitate identification of novel semiochemicals such as repellents. In addition, this may lead to the development of new or enhanced control tools for tsetse flies. Although this aspect of tsetse fly chemoreception is yet to be fully revealed, however, the availability of genomes of several tsetse fly species (IGGI 2014; Attardo et al. 2019), have aided annotations of chemosensory genes in the tsetse flies including those associated with chemoreception (Obiero et al. 2014; Macharia et al. 2016). This has also led to subsequent studies which are starting to unravel the sensory architecture of tsetse flies (including sensillum types, receptors, neurons, proteins, enzymes and genes), and their properties and functions (reviewed in Getahun et al. 2022b). Such molecular and chemosensory biology-assisted methods in combination with machine learning tools represent next generation approaches to isolation, identification, functional characterization and optimization of repellents and other semiochemicals for the control of tsetse flies.

## Data Availability

No datasets were generated or analysed during the current study.
